# Cerebral versus cortical visual impairment: eliminating the conflict and renewing the terminology

**DOI:** 10.1186/s12886-024-03469-8

**Published:** 2024-05-16

**Authors:** Marcelo Fernandes Costa

**Affiliations:** 1 Laboratório de Psicofísica e Eletrofisiologia Visual Clínica, Departamento de Psicologia Experimental, Instituto de Psicologia, Universidade de São Paulo, Av. Prof. Mello Moraes 1721 Cidade Universitária, São Paulo, 05508-030 SP Brasil; 2 Núcleo de Neurociências Aplicada, Faculdade de Medicina, Universidade de São Paulo, São Paulo, SP Brasil

**Keywords:** Cortical visual impairment, Cerebral visual impairment, Terminology proposal, Oculomotor disorders, High-order visual function, Diagnostics, Etiology

## Abstract

The inconsistency in terminology for Cortical Visual Impairment or Cerebral Visual Impairment presents challenges: (1) different levels of changes in visual pathway and other cerebral areas do not allow discrimination; (2) different visual and oculomotor aspects are not adequately considered. We open a debate to consider a more appropriate diagnosis.

## Background

The terminology of a condition is an important process by which we conceptualize and name things and events to be able to use this information cognitively. Forming concepts is thus of profound importance for the understanding of what we are dealing with. Recently, I have been concerned about the terminological variability with which a visual disease condition has been encountered in the literature. This condition is one in which visual impairments are not clinically detectable as being of ocular/ophthalmological origin. If not of ocular origin, there is the possibility that visual impairments are related to cortical changes that affect the primary visual pathway or higher areas, non-cortical areas such as thalamic and cerebellar lesions, as well as lesions in which more than one of these areas is affected. These different situations lead to functional differences that, in my opinion, have not been adequately considered in the literature.

These vision functional damages that are not of ocular origin can be found in the scientific literature as Cerebral Visual Impairment and Cortical Visual Impairment [1]. If the diversity of nomenclatures used as synonyms were not enough, while my arguments will demonstrate that they are different diseases, both can be expressed by the same acronym CVI. Also, there is no agreement between the ocular manifestation involved in the CVI diagnosis. In a stricter sense, CVI is related exclusively to post-chiasmatic lesions, which clinically do not generate identifiable changes, at least in the initial years after the injury. Changes due to retrograde degeneration may appear later and, thus, the presence of changes in higher sensitivity measurements such as OCT are sometimes identified [2]. Thus, the presence of small ocular changes such as temporal pallor of the papilla would prevent the use of the term CVI. The diagnosis of CVI with mild ocular changes may perhaps be permitted with recent technological advances in diagnosis and the term may be understood in a lax sense.

Cerebral Visual Impairment (CVI) stands as a prominent cause of low vision in the developed world, contributing to 27% of visual impairments in children [3]. Acquired forms may arise prenatally (such as intrauterine infections), perinatally (prematurity, ischemic brain injury), and postnatally (including hypoglycemia or meningitis). Perinatal issues constitute the most common causes of acquired CVI, accounting for two-thirds of cases. Four known CVI-associated genes, namely AHDC1, NGLY1, NR2F1, and PGAP1 [3], and other 19 candidate genes for CVI have been proposed.

Clinicians working with CVI children can appreciate the multifaceted challenges associated with the diagnosis, treatment, and management of these patients. In this article, we intend to present the different terminologies used, the epistemological implications that each one carries, and, finally, to propose a new terminology that allows identifying both in general and in a more specific way the different cases.

## Cortical visual impairment

The term Cortical Visual Impairment is defined as a bilateral loss of vision, normal pupillary response, and a clinical eye examination that shows no other abnormalities [4]. A more restrictive usage of terminology implies that the “cortical” refers to the white and gray matter of the brain [5]. This means that other structures such as the anterior part of the primary visual pathway, diencephalic, brainstem, and cerebellum are not affected. Considering that the primary cause of CVI is perinatal anoxia and periventricular leukomalacia, it is very unlikely that these insults are located only in the cortical layer of the encephalon [6].

Nonetheless, it is not difficult to find in the literature the use of CVI in which other alterations of the central nervous system are associated, such as vestibular, ocular motility, and optical atrophy [7].

## Cerebral visual impairment

Suppose we adhere to the logic that cortical visual impairment arises from changes in the cortex, while cerebral visual impairment results from lesions extending beyond cortical areas to affect subcortical and cerebellar structures. In that case, the literature does not consistently align with this reasoning. The investigation led by van Genderen and colleagues [8] examined children with CVI despite having good visual acuity. Some children had a visual acuity as high as 0.8. On the other hand, a significant number of these children with CVI face challenges in moving their eyes, posing a crucial issue that persists in clinical assessments. Two prevalent neuro-ophthalmological indicators of CVI include a persistent exotropia and often a horizontal tonic ocular deviation accompanied by a constant, tonic ipsiversive turn of the head [9]. These are examples of the interchangeable use of cortical/cerebral terminology for visual impairments contributes to an absence of a precise terminology that has a significative impact. A clear example is Cerebral Visual Impairment for children with good vision [8].

## A new terminology proposal

The need for a terminological readjustment has already been expressed by other authors in previous works [10]. We are proposing a terminology that integrates the anatomical and physiological characteristics that are presented in the different studies analyzed. At the same time, we seek to contemplate the visual functional aspects that are often affected in this population. Therefore, Central Visual Impairment (CVI) should be used as a Major Diagnostic Term. Under this nonspecific condition, we include Cortical Visual Impairment (termed CoVI) has its definition linked to deficits in visual functions exclusively due to lesions in the primary visual pathway, the visual cortex, and associated visual areas. Cerebral Visual Impairment (termed CeVI) should be used when, in addition to cortical visual impairments, other brain areas such as the cerebellum, brainstem, or thalamus are also affected. This evidence came from the presence of strabismus, nystagmus, and vestibule-ocular, pupillary, or accommodative impairments.

The measurement of visual functions such as visual acuity, contrast sensitivity, and visual field extent should, additionally, and according to the patient’s ability to understand and respond, perceptual visual functions including second-order motion perception, feature detection, visual attention, and memory.

## Conclusion

We presented a new terminology proposal called Central Visual Impairment (acronym CVI), in which the Cortical (acronym CoVI) and Cerebral Visual Impairment (acronym CeVI) can be accommodated accordingly, considering their multifactorial condition and great variability in clinical outcomes.

## Data Availability

No datasets were generated or analysed during the current study.

## Abbreviations

CVI: Cortical/ Cerebral/ Central Visual Impairment
CoVI: Cortical Visual Impairment
CeVI: Cerebral Visual Impairment
OCT: Optical Coherence Tomography
AHDC1 gene: AT-Hook DNA-Binding Motif-Containing Protein 1
NGLY1 gene: Peptide-N(4)-(N-Acetyl-Beta-Glucosaminyl)Asparagine Amidase
NR2F1 gene: Nuclear Receptor Subfamily 2 Group F Member 1
PGAP1 gene: Post-GPI Attachment to Proteins 1

## Declarations

### Author’s information

The author is the coordinator of the Central Visual Processing Ambulatory and the Clinical Visual Psychophysics and Electrophysiology Sector of the Experimental Psychology Department of the Psychology Institute of the University of São Paulo
